# Local Alignment Refinement Using Structural Assessment

**DOI:** 10.1371/journal.pone.0002645

**Published:** 2008-07-09

**Authors:** Pierre Chodanowski, Aurélien Grosdidier, Ernest Feytmans, Olivier Michielin

**Affiliations:** 1 Swiss Institute of Bioinformatics, Bâtiment Génopode, Lausanne, Switzerland; 2 Ludwig Institute for Cancer Research, Epalinges, Switzerland; 3 Centre Pluridisciplinaire d'Oncologie, Centre Hospitalier Universitaire Vaudois, Lausanne, Switzerland; University of Queensland, Australia

## Abstract

Homology modeling is the most commonly used technique to build a three-dimensional model for a protein sequence. It heavily relies on the quality of the sequence alignment between the protein to model and related proteins with a known three dimensional structure. Alignment quality can be assessed according to the physico-chemical properties of the three dimensional models it produces.

In this work, we introduce fifteen predictors designed to evaluate the properties of the models obtained for various alignments. They consist of an energy value obtained from different force fields (CHARMM, ProsaII or ANOLEA) computed on residue selected around misaligned regions. These predictors were evaluated on ten challenging test cases. For each target, all possible ungapped alignments are generated and their corresponding models are computed and evaluated.

The best predictor, retrieving the structural alignment for 9 out of 10 test cases, is based on the ANOLEA atomistic mean force potential and takes into account residues around misaligned secondary structure elements. The performance of the other predictors is significantly lower. This work shows that substantial improvement in local alignments can be obtained by careful assessment of the local structure of the resulting models.

## Introduction

The three-dimensional structure of proteins is central to many applications, such as structure-function studies, site-directed mutagenesis, or structure based design of active compounds. Since the creation of the Protein Data Bank [1], the number of protein structures solved by experimental techniques have grown exponentially, with more than 38000 protein structures available today (as of 7 September 2006). Despite this strong experimental effort, it represents hardly above 1% of the number of proteins of the SwissProt plus TrEMBL databases (SwissProt release 50.6 of 5 September 2006; TrEMBL release 33.6 of 5 September 2006). Interestingly, only 945 different folds are currently represented in the PDB, as reported by the SCOP classification [2]. This can be compared to the 4000 different folds that are predicted to be present in the proteome [3]. Homology modeling methods, in which a structural model for a protein with a known sequence (the target) is generated using experimental structures of related proteins (templates), provide a way to close the gap between the large number of known sequences and the limited number of related structures. Homology modeling methods are based on the fact that proteins with a detectable degree of sequence identity associated with the conservation of topology and function are very likely to share the same fold [2], [4].

To this day, homology modeling methods represent one of the most reliable approache to generate a structural model for a protein sequence [5], when at least one suitable template is available. From the fifth Critical Assessment of techniques for protein Structure Prediction (CASP5) experiments [6], it appears that the critical steps to obtain a good model are: 1) the selection of the template, 2) the alignment between the target and templates sequences, 3) the modeling of regions not present or structurally different from those in the template and 4) the modeling of side chains. Among these, the second step is the most critical because a wrong alignment between the target and templates will systematically lead to misfolded models [7]. When the sequence identity between a target and its template(s) is above 50%, a pair wise alignment is usually correct and the resulting model quality is comparable to low resolution crystal structures or medium-resolution NMR structures [8]. When the sequence identity ranges from 25% to 50%, serious errors are prone to appear in the alignment. Below 25%, the quality of an automated alignment is usually not sufficient to build an accurate model and human expertise and/or experimental data are needed. Multiple sequence alignments can certainly help stretching the range of usable sequence identity, but they are essentially subject to the same limitations.

The local sequence identity is usually variable along the alignment between the target and its template. This often leads to situations where two regions of the alignment are easy to align, but are separated by a short stretch where the sequence identity is locally low, and for which a relatively small number of alternative alignments have to be considered. It was proposed to evaluate these alternative alignments through their corresponding homology models [see 9], [10], [11], [12 and see below]. In such approaches, errors in the initial sequence alignment might be identified subject to two conditions. First, it should be possible to obtain reliable models for each alternative alignment. Second, as pointed out by John et al. [9], a discriminative scoring function should be available to point out the model obtained from the correct alignment among the models obtained from the wrong ones. Model quality can be assessed using various techniques, like geometric or energetic criteria derived from known protein structures. Among the formers, PROCHECK [13] is widely used to scan a model for unlikely bonds, angles and dihedrals values and for the solvent accessible surface of amino acids. While such criteria are useful to describe the quality of a protein structure locally, i.e. at the residue level, their ability to recognize a misfolded model is limited [14]. To this aim, methods based on a mean force potential (MFP) describing the free energy of interaction between atoms or residues have been developed. Verify3D [15], using statistical preference of amino acids for their environment, could be considered a precursor of MFP methods. ProsaII [16] is a residue based MFP using local and non-local interactions. ANOLEA (17, http://protein.bio.puc.cl/cardex/software/index.html) is an atom-based MFP, where only non-local interactions are taken into account.

Scoring schemes relying on physics-based energies haves been used to discriminate between native and near-native structures [18], [19], [20]. These energies are computed from molecular mechanics energy functions with solvation models [18], [21] but do not include entropic terms. Machine learning-based methods combine scores from physics-based energies and statistical potentials. They include Neural Network [22], Genetic Algorithm [23] and Support Vector Machine [14].

A recent benchmark involving physics-based scoring functions, MFP and machine learning based approaches pointed out that structural information is very informative when evaluating the quality of an alignment [14], [24]. The need to restrict scoring functions around the region to optimize has also been suggested [22], [25].

In order to evaluate the quality of models, fifteen predictors were defined in this article. Each of them consists in an energy function computed on a wide or narrow residue selection around misaligned regions. For each alternative alignment, the MODELLER program [11] is used to build an ensemble of one hundred models, all solutions of the same distance geometry problem, but with different initial conditions for the molecular dynamics optimization step. These models sample the conformational space allowed by the alignment derived restraints used during the dynamics. The local quality of the models evaluated by the predictors is used to identify the optimal alignment among all possible ungapped alignments.

Five energies functions were investigated: the standard CHARMM energy [27] with two different dielectric constants, the CHARMM energy including the solvation free energy computed using the Generalized Born model (GBMV2 [28], [29]), and two MFP, ProsaII and ANOLEA.

Three different residue selections around the misaligned region were investigated, taking into account either all the residues of the model, or the residues contained in the misaligned region plus their close neighbors, or the residues in the misaligned regions belonging to a secondary structure element plus their close neighbors.

The fifteen predictors were assessed on ten challenging local alignments optimization problems of both α helix and β sheets, among which challenging CASP cases with no sequence identity between the template and the target. The best predictor was able to retrieve the structural alignment for 9 out of the 10 test cases. It is based on the ANOLEA energy computed on a subset of residues around the misaligned secondary structure element. The success rate of predictors based on ProsaII is at most 60%, and predictors based on the CHARMM 19 or 22 energies, with or without the solvation free energy, remain lower than 30%.

In what follows, we first describe the ten local alignments optimization problems selected to assess our method. Second, the generation and scoring of alignments is presented step by step. Third, the performance of the various predictors is presented and detailed for a challenging CASP5 target. Finally, the physical ground of this approach is discussed.

## Materials and Methods

**Figure 1:**
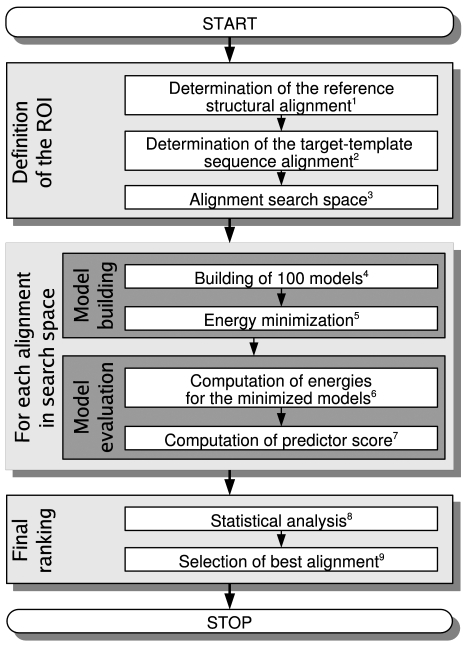
Flowchart of the method. A reference structural alignment of the target and the template is generated by the MALIGN3D command in MODELLER [11] (step 1). The initial target-template sequence alignment is realized by T_COFFEE (step 2). The regions of interest (ROI), defined as misaligned secondary structure elements together with their adjacent loops, are identified by comparison of the initial target-template sequence alignment with the reference structural alignment. A set of alignments to evaluate is generated using an exhaustive ungapped search in the ROI (step 3). Hundred models for each alignment are built using MODELLER (step 4). For each model, an energy minimization is done in vacuum using CHARMM (step 5). The energy for the minimized models is calculated (step 6). The secondary structure is assigned with DSSP [24] and the predictor's scores are calculated (step 7). After all alignments are processed, a statistical analysis using the statistical package R (http://www.R-project.org) is further performed on the predictor to associate a degree of confidence to the prediction (step 8) and the best alignment is determined (step 9). See Materials and Methods for details.

The main steps of our approach are outlined in Figure 1 and detailed below.

### Identification of the region of interest (ROI)

**Figure 2:**
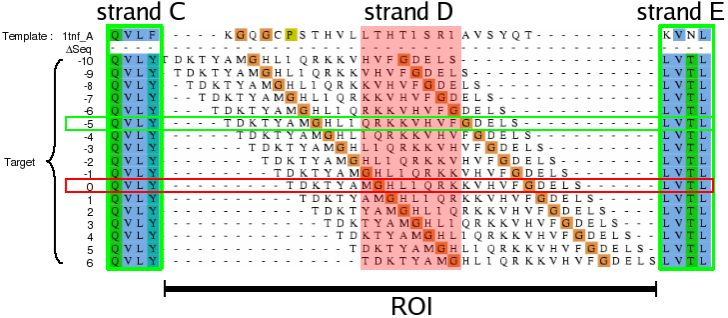
Illustration of the sliding window method for the case of hBAFF strand D. The initial sequence based alignment of hBAFF sequence with 1tnf sequence was realized with T_COFFEE. The region of interest (ROI) is defined as the misaligned secondary structure element (strand D) together with its adjacent loops. All residues in the ROI are grouped in one ungapped block. The method consists in sliding this block through the window (red rectangle) defined by strand D of the 1tnf (chain A) template. The explored alignments were identified by their sequence offset (Δseq) to the structural alignment. It corresponds to a sequence offset to the structural alignment of Δseq = −5 (green horizontal rectangle). The structural alignment is right shifted by five position (Δseq = 0, red horizontal rectangle).

For each test case (see below), the structural alignment between the target and the template was computed using the MALIGN3D routine in the MODELLER. This alignment was used as a reference to which the alternative alignments are compared. An initial sequence-based sequence alignment was computed with T-Coffee [30], using default parameters. In the selected test cases (see below), comparison of the sequence-based alignment and the structural alignment revealed several discrepancies observed in both loop regions and secondary structure elements (SSE). Due to their high sequence variability and intrinsic flexibility, loops are usually not well predicted using homology based approaches and are better suited for *ab initio* methods [31]. For this reason, this study focuses on local alignment optimization of SSE without a direct optimization of loop alignments. The ROI was defined as a misaligned region containing a SSE limited at the N and C terminal part by two unambiguously aligned regions or by one unambiguous region and a chain termini, see Figure 2.

### Alignment search space

The residues in the ROI were grouped in one ungapped mobile block of residues containing the misaligned SSE and its adjacent loops, if any. All possible ungapped alignments were generated by shifting this block along the template sequence (Fig. 2), with the constraint that the SSE of the target sequence always contained a constant number of amino acids. This procedure is referred to as “sliding window” below. Each alternative alignment was identified by the offset, Δseq, between the position of the mobile block in the alternative alignment compared to its position in the reference structural alignment; by definition, the structural alignment is characterized by Δseq = 0, a negative Δseq value is used when the amino acids of the target are moved toward the N-terminal region of the template, and a positive Δseq when moved to the C-terminal.

### Model building

From each of the alternative alignments explored by the sliding window search described above, 100 models were built by the MODEL homology modeling routine of MODELLER 6.2. Disulfide bonds were explicitly defined, and default parameters were used. To build models, MODELLER satisfies spatial restraints derived from the sequence alignment between the target and its templates. The optimization is based on MD simulations; different models can be generated using different random seeds for the assignment of the initial random velocities.

Each model generated by MODELLER was energy minimized using the CHARMM program with the CHARMM19 force field [32], [33], a dielectric constant of 1 and a 20 Å cutoff. This minimization consisted in 30 steps of Steepest Descent, followed by 30 steps of Adopted Basis Newton-Raphson. Positions of the Cα atoms were constrained using mass weighted harmonic forces constant of 10 kcal/(mol Å^2^) that were present during the entire minimization.

### Model evaluation using predictors

The models obtained after energy minimization were evaluated by means of various “predictors”. A predictor is defined as an energy function combined with a selection of residue. First, the different energy terms are calculated for each selected residue taking into account the complete environment, and then the sum of the energy of the selected subset of residues (see below) is assigned to the model. When all models generated for a given alignment have been evaluated, the predictor score distribution of the formers is assigned to the latter. When all alternative alignments have been evaluated, a statistical assessment is performed to compare the distribution of their predictor scores.

A total of fifteen different, yet closely related, predictors were assessed, taking into account five different energy functions (CHARMM ε = 1, CHARMM ε = 4, CHARMM GBMV2, ProsaII and ANOLEA) and three different selections of residues (All, ROI, SSE) corresponding to wide or narrow region around the misaligned SSE, see below.

### Energy functions

Five different energy types were used to evaluate the structural models.

### CHARMM energies

The CHARMM program was used to compute the energy of the selected residues using the CHARMM19 or the CHARMM 22 force field. The electrostatic contribution was computed in three different ways: 1) using a distance-independent dielectric value of 1 (vacuum), referred to as CDIE ε = 1, 2) using a distance-dependent dielectric value (simple electrostatic screening), referred to as RDIE ε = 4, and 3) using the Generalized Born using Molecular Volume analytical method 2 (electrostatic solvation energy), referred to as GBMV2 below.

### ProsaII score

The ProsaII MFP [16] was used to compute the energy of the selected residues using a window of 1 residue.

### ANOLEA energy

Similarly, the ANOLEA MFP was used to compute the energy of the selected residues using the recommended averaging over five contiguous residues.

### Residues selections

Three different residue selections were assessed, from wider to narrower around the ROI.

First, the *All* selection takes into account all residues of the model.

Second, the *ROI* selection contains two subsets of residues. The first subset contains all residues of the ROI. The second subset contains all the residues surrounding the first subset with a maximum distance threshold between heavy atoms. The optimal distance threshold was investigated (see results). Since the second subset varies from model to model, only residues that meet the distance threshold in at least 50% of the models were considered and used to assess the energy of the 100 models.

Third, the *SSE* selection also contains two subsets of residues. The first subset contains only the residues of the SSE of the ROI. The second subset contains all the residues surrounding the first subset with a maximum distance threshold between heavy atoms. Again, only residues that meet the distance threshold in at least 50% of the models were considered.

### Statistical analysis

A statistical analysis of the distributions of predictor scores was carried out to check whether the score distributions of the predictors computed for each alignment are significantly different. A Kolmogorov-Smirnov test showed that distributions were not normal and a Bartlett's test revealed that their variances are also different (data not shown). The conditions were not met to use a Student's t-test and a non-parametric rank-based Wilcoxon test was performed instead, using a confidence threshold α of 0.05. The statistical package R (http://www.r-project.org) was used to carry out the tests.

### Choice of test cases

Challenging alignment optimization problems were selected from the literature according to the following criteria: the global sequence identity between the target and its template must be lower than 35%, a crystal structure must be available for the target, and the sequence based and structural alignments must differ in one or more regions encompassing secondary structure elements (SSEs). Proteins with various folds were selected: α helices (α), only β-strands (β) and both α and β (α/β). We chose a CASP1 target: the human eosinophil-derived neurotoxin (EDN) [34], five CASP5 targets: T0141, T0143, T0151, T0169 and T0178, [35], a low sequence identity pair of hemoglobin protein (1ash-1flp) and the homotrimeric human B cell activating factor (hBAFF) protein, which is a member of the tumor necrosis factor ligands (TNFL) family. For EDN, the initial sequence alignment between the target and its template was that proposed by Sali [34]. All test cases are listed in Table 1.

**Table 1:** List of the test cases.

*Target*	*Length in residues*	*Fold* a	*Template* b	*Global seq id* c * (%)*	*SSE seq id in ROI* d * (%)*	*SSE content and limits in ROI* e	*Residues in ROI* f	*Initial ΔSeq* g
hBAFF	432	β homo trimer	1tnf, A,B,C	22	38	β-strandD (M208-K215) and (M352-K359) and (M496-K503)	D203-L224 D347-L368 D491-L512	−5 −5 −5
1flp	142	α	1ash	13	13	α helix1 (A4-A19)	S1-A20	5
1flp	142	α	1ash	13	6	α helix7 (A103-Y120)	G100-G121	−3
EDN	134	α / β	7rsa	33	56	α helix1 (W7-H15)	K1-S20	6
T0141	187	α / β	1aro, L	14	33	β-strand10 (E116-E118)	C108-A124	6
T0143	216	α / β	1agj, A	27	10	α helix6 (E200-N209)	N199-A216	−4
T0151	106	α / β	1eyg, D	33	0	β-strand7 (E105-P108)	D104-S123	−4
T0169	156	α / β	1l0c, A	17	17	β-strand5 (R104-T109)	R100-V112	−3
T0178	219	α / β	1jcj, A	27	50	β-strand8 (R300-T303)	D299-S304	−12
T0178	219	α / β	1jcj, A	27	6	α helix7 (Y201-R213)	S198-A218	3

a Fold of the target according to SCOP.

b PDB code of the template and its chain identifier, when present.

c Structure-based global sequence identity between the target and the template.

d Structure-based sequence identity between the target and the template of the secondary structure element (SSE) in the region of interest (ROI).

e Type of SSE in the ROI with its limits on the target sequence. The SSE limits on the target sequence are deduced from the template SSE using the alignment correspondence.

f Limits of the ROI on the target sequence, (the ROI includes the residues of the SSE plus the residues in the adjacent loops).

g The offset of the initial target-template alignment (see Material and Methods) from the structural alignment is quantified by ΔSeq. The reference is the structural alignment (ΔSeq = 0). A shift of the target SSE sequence to the C-terminal or N-terminal part has positive or negative value, respectively.

## Results

This article addresses the question of the local optimization of the sequence alignment between a target sequence and its corresponding template, a critical problem in homology modeling. This optimization was carried out by generating all possible ungapped alignments, for which a score is assigned according to the quality of their corresponding models. This score is based on different subsets of particular pair wise energy types, termed “predictors”. The method is outlined in Figure 1 and detailed in Material and Methods. The ROI chosen for each test case, and the corresponding alignment search space are presented first. Second, the variability between models is presented, as well as the impact of the energy minimization. The results for all predictors are then discussed, followed by an insight into the most efficient one. Finally, an illustrative example is detailed.

### Selected ROI

**Figure 3:**
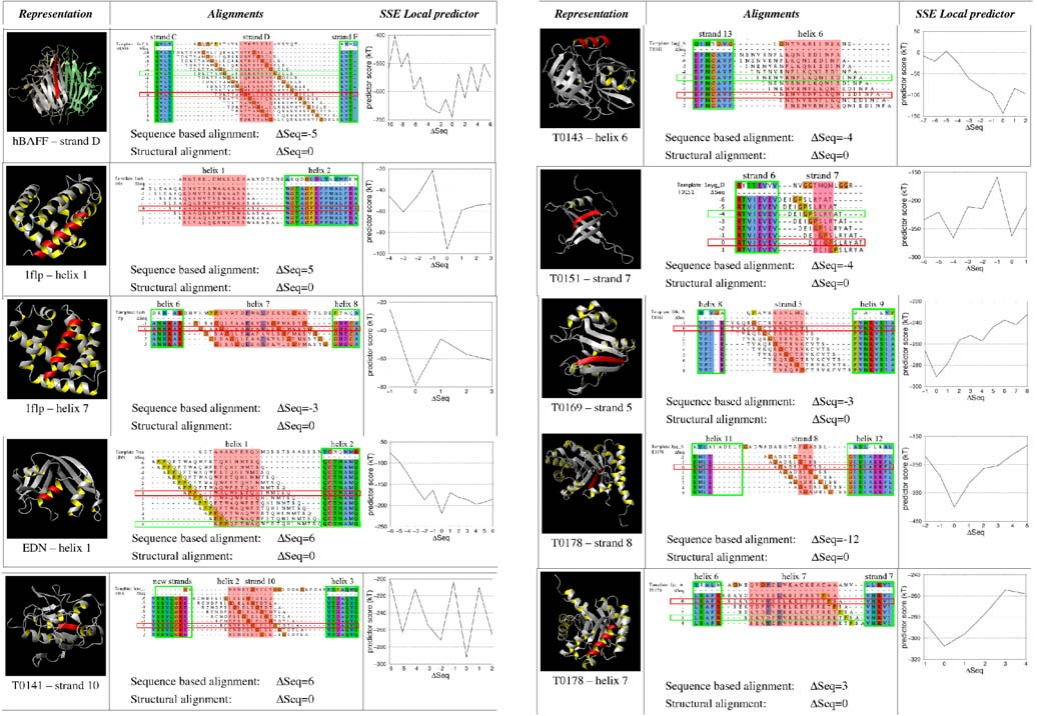
For each test case, the SSE in the ROI is colored in red on a protein ribbon representation and is defined by a red rectangle in the alignments. The different alignments are represented and the corresponding score using the ANOLEA/SSE Local predictor is plotted. In T0141 test case, the “new strands” label in the alignment picture represents the position of a beta finger present in the structure of T0141 but absent in the 1aro_L template.

A single ROI was selected for hBAFF (strand D), EDN (helix 1), T0141 (strand 10), T0143 (helix 6), T0151 (strand 7) and T0169 (strand 5). Two ROI distant in the sequence space were independently refined for 1flp (helix 7 then helix 1) and T0178 (strand 8 and helix 7). The alignment search space for each test case is reported in Figure 3. The global sequence identities between targets and their templates range from 13 to 33%, and the local sequence identities of the SSE in the ROI range from 0 to 56%, see Table 1. For each ROI, the offset between the sequence alignment and the structural alignment (Δseq) is given in the last column.

### Models building

**Figure 4:**
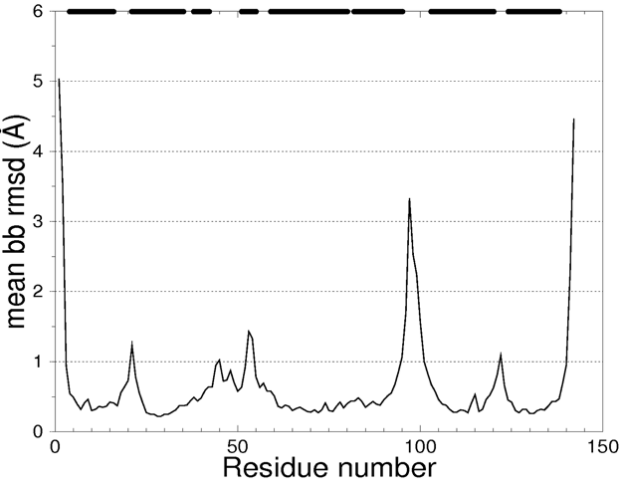
Mean backbone (bb) RMSD per residue for 100 models of 1flp h1 testcase for Δseq = 0. Secondary structure elements limits are indicated by horizontal bars on the upper x axis. Regions of high variability correspond to loops.

The structural variability between 100 models computed from the structural alignment using MODELLER and energy minimized using CHARMM is illustrated for the 1flp helix 1 test case, using the per residue backbone RMSD after optimal superimposition of the entire structure (Figure 4). As expected, the RMSD is higher in loops than in structured regions. This comes from the fact that the loops are less confined by alignment derived restraints, due to their lower sequence identity as well as their inherent structural flexibility compared to secondary structure elements that are stabilized by well known hydrogen bond interaction.

### Model minimization

**Figure 5:**
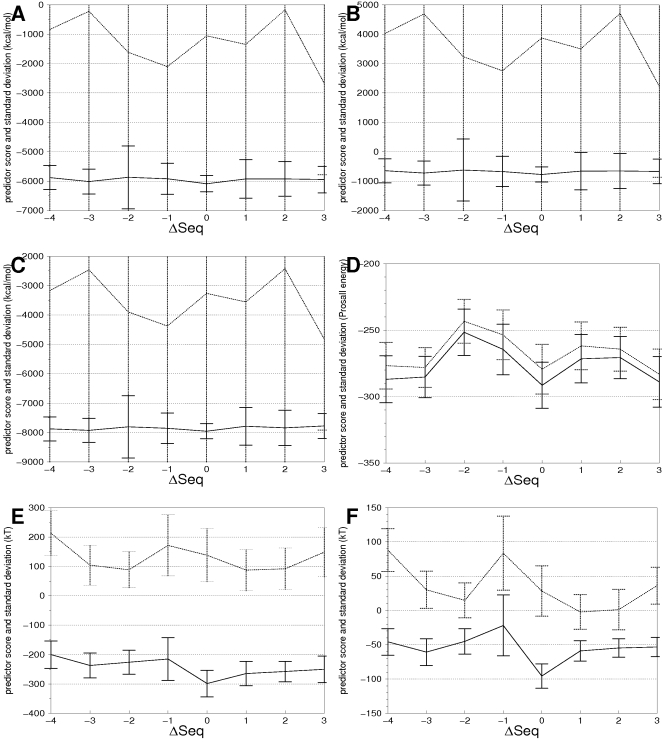
Influence of energy minimization for six different predictors CDIE/All (A), RDIE/All (B), GBMV2/All (C), ProsaII/All (D), ANOLEA/All (E) and ANOLEA/SSE (F) applied to the test case 1flp helix1. The six corresponding profiles and their standard deviation are shown for minimized and non minimized models, with plain and dotted lines, respectively.

The energy of minimized models is, as expected, both much lower and less variable, as illustrated in Figure 5 for the 1flp helix 1 case. An important decrease in both the energy and its variability for CHARMM-based predictors was observed after energy minimization (Fig. 5A–C). ANOLEA-based predictors are also significantly influenced by energy minimization (Fig. 5E), while ProsaII-based predictors are marginally impacted (Fig. 5D), as expected for a residue based force field.

The correct alignment for 1flp helix 1 (Figure 5F) was obtained using the *ANOLEA/SSE* predictor from minimized models (p-value = 1.6⋅10^−23^), whereas it was not retrieved when non minimized models were used. For other test cases, the removal of this minimization step lead to less discriminative (as reflected by higher p-values), or even wrong predictions (data not shown). This supports that energy minimized models not only help in reducing the variance of the energy but also change the predictor landscape, allowing better predictions to be made. The energy of models was thus always minimized before evaluation by the predictors.

### Evaluation of the predictors

The results obtained with the fifteen predictors over the entire test set are summarized in Table 2 and 3 and detailed below.

**Table 2:** Summary of alignment refinement results for our set of test cases using two enthalpic scoring functions and three different residues selections.

*Target*	*SSE in the ROI* a	*Initial* b * ΔSeq*	*CHARMM19*									*CHARMM22*								
			*CDIE ε = 1*			*RDIE ε = 4*			*GB*			*CDIE ε = 1*			*RDIE ε = 4*			*GB*		
			*All*	*ROI*	*SSE*	*All*	*ROI*	*SSE*	*All*	*ROI*	*SSE*	*All*	*ROI*	*SSE*	*All*	*ROI*	*SSE*	*All*	*ROI*	*SSE*
1kxg (hBAFF)	Strand D	−5	**F**	*s*	**S**	*f*	*s*	*s*	*f*	*s*	**S**	*f*	*f*	*f*	*f*	*f*	*f*	*f*	*f*	*F*
1flp	Helix 1	5	**S**	*f*	**S**	*s*	*s*	*s*	*s*	*f*	**S**	**S**	**F**	**F**	*s*	**F**	**S**	*f*	**F**	**F**
1flp	Helix 7	−3	**F**	**F**	**F**	**F**	**F**	**F**	*s*	**F**	**F**	*f*	**F**	**F**	*s*	**S**	**S**	**S**	**S**	**S**
1gqv (EDN)	Helix 1	6	*f*	*f*	*f*	**F**	*f*	*f*	**F**	*f*	*f*	*f*	**F**	**S**	*f*	**S**	**S**	*f*	**S**	**S**
1j3g (T0141)	Strand 10	6	*s*	**S**	**S**	*s*	**S**	**S**	*s*	**S**	**S**	**S**	**F**	*f*	*f*	*f*	**S**	*s*	**F**	*f*
1qy6 (T0143)	Helix 6	−4	*f*	*f*	*f*	*f*	*f*	*f*	*f*	*f*	*f*	**F**	**F**	**F**	**F**	f	**F**	**F**	**F**	**F**
1ue6 (T0151)	Strand 5	−4	**F**	**F**	**F**	**F**	**F**	**F**	**F**	**F**	**F**	**F**	*f*	**F**	*f*	**F**	**F**	*f*	*f*	*f*
1mk4 (T0169)	Strand 5	−3	*s*	*s*	*s*	*s*	*S*	*s*	*s*	*s*	*s*	*f*	**F**	**F**	**S**	*f*	**F**	*f*	**F**	**F**
1mhz (T0178)	Strand 8	−12	*s*	*f*	*f*	*s*	*f*	*f*	*s*	*f*	*f*	**S**	**S**	**S**	*s*	**S**	**S**	**S**	**S**	**S**
1mhz (T0178)	Helix 7	−3	**F**	**F**	**F**	**F**	**F**	**F**	**F**	**F**	**F**	**S**	**S**	*f*	**S**	**S**	*s*	**S**	**S**	*f*

F stands for failure and S for success. When the p-value associated with the prediction is significant, the outcome is formatted in bold character, otherwise in lowercase italic.

a Secondary structure element (SSE) in the region of interest (ROI) (see Table 1 for more details about the ROI).

b Offset between the initial target-template alignment (see Material and Methods) and the structural alignment. The reference is the structural alignment (ΔSeq = 0). A shift of the target SSE sequence to the C-terminal part (N-terminal) has negative (positive) value.

**Table 3:** 

*Target*	*SSE in the ROI* a	*Initial* b * ΔSeq*	*Prosa II*			*ANOLEA*		
			*All*	*ROI*	*SSE*	*All* c	*ROI* d	*SSE* e
1kxg (hBAFF)	Strand D	−5	*f* 9.0⋅10^−1^	**F** 3.4⋅10^−8^	**F** 1.2⋅10^−4^	**S** 3.8⋅10^−2^	*s* 7.4⋅10^−2^	**S** 9.6⋅10^−3^
1flp	Helix 1	5	*s* 4.8⋅10^−1^	**S** 2.4⋅10^−2^	*f* 2.0⋅10^−6^	**S** 1.2⋅10^−8^	**S** 2.4⋅10^−20^	**S** 1.6⋅10^−23^
1flp	Helix 7	−3	**S** 1.2⋅10^−28^	**S** 2.8⋅10^−34^	**S** 2.6⋅10^−34^	*s* 8.7⋅10^−1^	**S** 6.1⋅10^−3^	**S** 6.5⋅10^−13^
1gqv (EDN)	Helix 1	6	**S** 4.1⋅10^−3^	**S** 8.0⋅10^−25^	**S** 7.2⋅10^−22^	*f* 8.0⋅10^−1^	**F** 9.5⋅10^−3^	**S** 4.1⋅10^−11^
1j3g (T0141)	Strand 10	6	*s* 5.7⋅10^−2^	*s* 3.7⋅10^−1^	*s* 1.6⋅10^−1^	*s* 4.8⋅10^−1^	**S** 3.0⋅10^−2^	**S** 2.7⋅10^−3^
1qy6 (T0143)	Helix 6	−4	**S** 9.5⋅10^−30^	**S** 5.3⋅10^−15^	**S** 2.6⋅10^−34^	**S** 3.4⋅10^−8^	**S** 7.0⋅10^−19^	**S** 1.1⋅10^−22^
1ue6 (T0151)	Strand 5	−4	*s* 5.5⋅10^−1^	*s* 5.6⋅10^−1^	**S** 2.0⋅10^−22^	*f* 9.4⋅10^−1^	**F** 1.6⋅10^−19^	*s* 3.9⋅10^−1^
1mk4 (T0169)	Strand 5	−3	**S** 3.0⋅10^−5^	**S** 3.9⋅10^−7^	**S** 5.4⋅10^−30^	*s* 3.0⋅10^−1^	*s* 3.3⋅10^−1^	**S** 5.0⋅10^−2^
1mhz (T0178)	Strand 8	−12	**F** 4.1⋅10^−2^	**F** 9.9⋅10^−7^	**F** 1.1⋅10^−3^	**S** 1.3⋅10^−13^	**S** 4.2⋅10^−32^	**S** 2.2⋅10^−32^
1mhz (T0178)	Helix 7	−3	*f* 1.2⋅10^−6^	*s* 4.8⋅10^−1^	*f* 6.3⋅10^−34^	*s* 3.5⋅10^−1^	*s* 8.9⋅10^−1^	**S** 2.0⋅10^−2^

Summary of alignment refinement results for our set of test cases using two scoring functions based on potential of mean force combined with three different residues selections, as well as the associated p-values (see text for details). F stands for failure and S for success. When the p-value associated with the prediction is significant, the outcome is formatted in bold character, otherwise in lowercase italic.

a Secondary structure element (SSE) in the region of interest (ROI) (see Table 1 for more details about the ROI).

b Offset between the initial target-template alignment (see Material and Methods) and the structural alignment. The reference is the structural alignment (ΔSeq = 0). A shift of the target SSE sequence to the C-terminal part (N-terminal) has negative (positive) value.

c Prediction based on *All* residues selection.

d Prediction based on *ROI Local* residues selection.

e Prediction based on *SSE Local* residues selection.

### Comparison of the different energy types

Predictions based on the CHARMM 19 and 22 force field (CDIE, RDIE, GBMV2) show that the different level of approximation to treat the solvent has little impact, with a maximum success rate of 30%, whatever the residues selections taken into account. Due to the poor performance of these predictors, the p-values are not reported in Table 2 and their results are not discussed further. The performance of other predictors is shown in Table 3.

The reliability of ProsaII-based predictors is marginally impacted by the subset of residues taken into account, as reflected by their success rates: 60% for *All*, 50% for *ROI* and 50% for *SSE*.

In contrast, the success rate of ANOLEA-based predictors increase as the subset of residues taken into account is narrowed around the SSE: 40% for *All*, 50% for *ROI,* and 90% for *SSE*. The *ANOLEA/SSE* predictor is thus the most reliable predictor among the fifteen predictors tested.

### Comparison of residue selection

Optimization of the selection of neighbors

**Figure 6:**
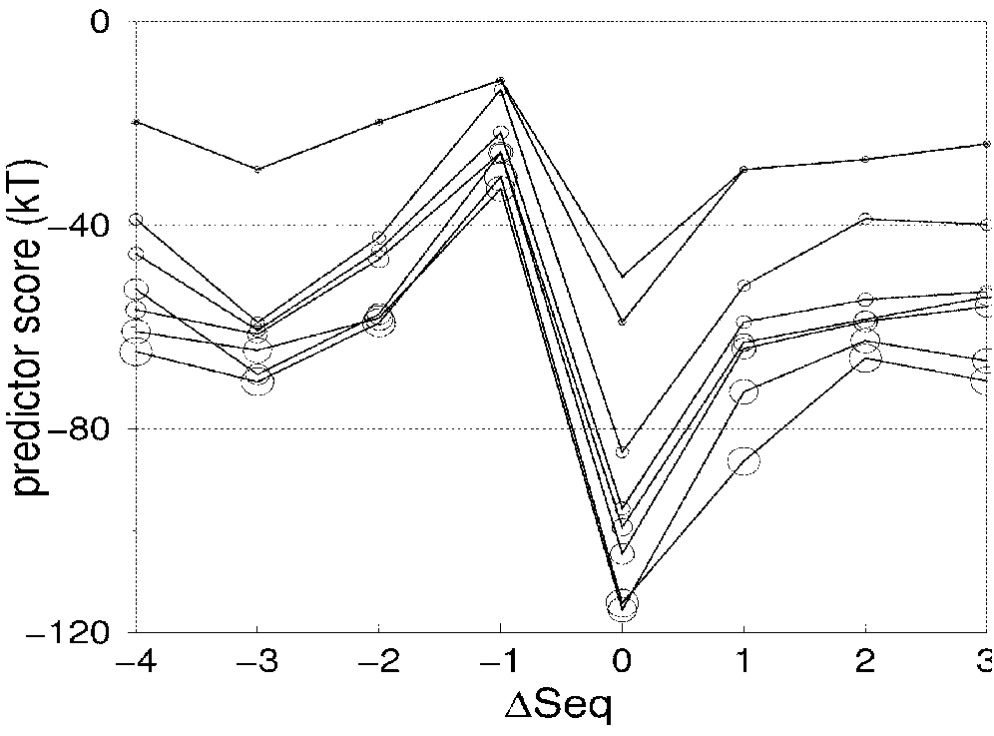
Optimization of the ANOLEA/SSE Local predictor specificity. The highest specificity is attained for an inter-residue distance cut-off of all pairs of heavy atoms of 4 Å as deduced from the scan by step of 0.5 Å from 2 to 6 Å for all the test cases. ANOLEA/SSE Local predictor scores for 1flp helix1 test case versus alignment offset to the structural alignment and for different inter-residue distance of all pairs of heavy atoms (from 2 Å (smallest sphere) to 6 Å (biggest sphere)).

The selections *ROI* and *SSE* include neighboring residues (see Material and Methods). Two residues are considered neighbors if, in at least 50% of the models generated, one of their heavy atom distances is smaller than 4 Å, in order to focus on the first shell of residues around the SSE. The importance of this cutoff was evaluated for the 1flp helix test case by exploring values ranging from 2 to 6 Å by step of 0.5 Å, in combination with the most successful predictor, *ANOLEA/SSE* (Figure 6). Interestingly, the structural alignment is identified for each cutoff value, and our approach appears to be robust regarding this parameter (data not shown). As mentioned above, an arbitrary value of 4 A was used for all calculations.

### Influence of the residue selection on the ANOLEA predictor

**Figure 7:**
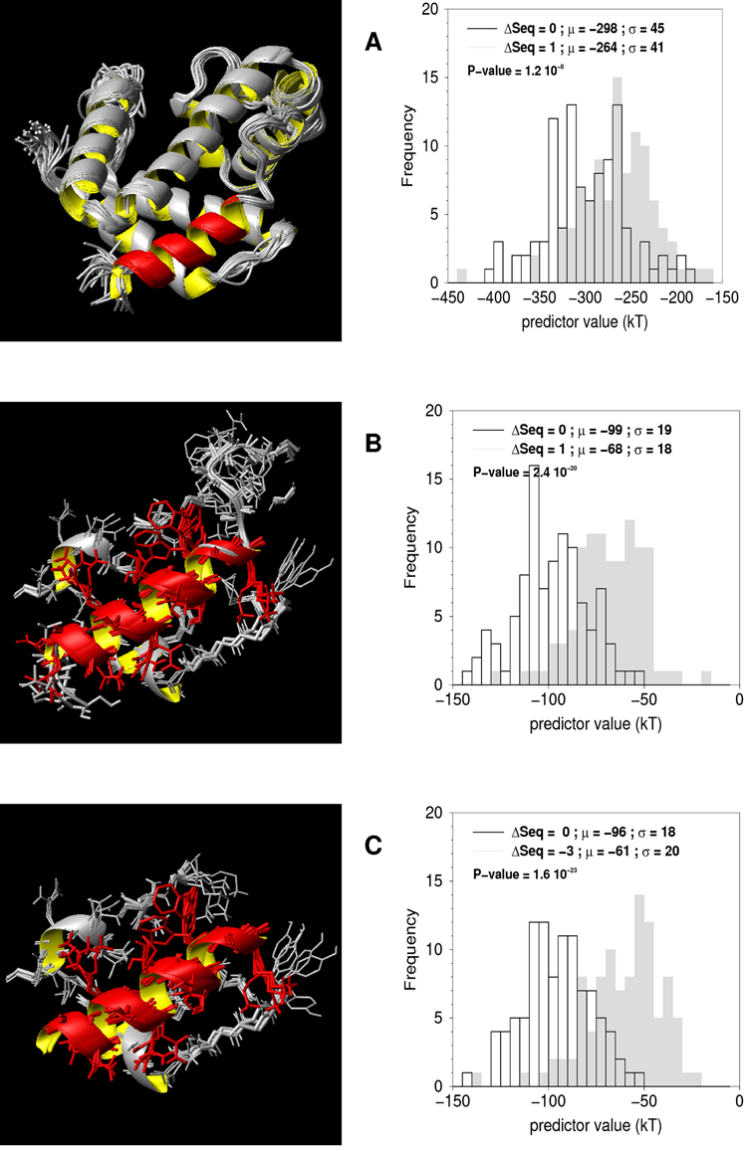
ANOLEA-based predictors' values distribution for the three different residues selections. A hundred models of 1flp helix1 test case are superimposed. Only parts of the protein involved in each selection are represented and the corresponding predictor values distribution are shown. The SSE in the ROI is represented in red. A, Predictor ANOLEA/All. B, Predictor ANOLEA/ROI Local. C, Predictor ANOLEA/SSE Local. The black and gray colored histograms represent the predictors values distributions for the lowest predictor scores (structural alignment, ∶seq = 0) and for the second lowest one, respectively. The bin width is set equal to the quarter of the variance (10 for A, 5 for B and C).

The structural variability between models resulted in a broad energy distributions for the *ANOLEA/All* predictor (Fig. 7A). In the figure, the two distributions with the lowest mean score values are shown. As can be seen, the distributions of predictor scores overlap. With the *ROI* selection, the variance arising from the conformational variability of residues not in the neighborhood of the ROI is removed (Fig. 7B). Compared to the *ANOLEA/All* selection, the distributions are thus narrower and better separated. These distributions are even sharper for the *ANOLEA/SSE* predictor because the variations caused by loops flanking the SSE in the ROI are also excluded. This is reflected by much lower p-values (Fig. 7C). A summary of the predictions based on ANOLEA and their associated p-values is presented in Table 3.

The *ANOLEA/All* predictor was able to identify unambiguously the structural alignments for hBAFF, 1flp (helix 1), T0143 and T0178 (strand 8). Associated p-values range from 3.8⋅10^−2^ to 1.3⋅10^−13^. The structural alignment was ambiguously identified for 1flp (helix 7), T0141 and T0169, as reflected by not statistically significant p-values. The prediction is wrong for EDN, T0151 and T0178 (helix 7).

The performance of the *ANOLEA/ROI* predictor is better than the *All* predictor, with associated p-values for successful prediction ranging from 3.0⋅10^−2^ to 4.2⋅10^−32^. Compared to the *ANOLEA/All* predictor, these lower p-values reflect a higher confidence as the residues selection is narrowed around the ROI.

The prediction results for the *ANOLEA/SSE* predictor are given in Table 3 and shown in Figure 3. The alignment corresponding to the structural alignment has the most favorable score in 9 out of the 10 test cases, associated with statistically significant p-values, ranging from 5.0⋅10^−2^ to 2.2⋅10^−32^. Among the three ANOLEA-based predictors, *ANOLEA/SSE* was found to be the most successful, and also lead systematically to statistically more significant p-values.

If Anolea/SSE and ProsaII/SSE predictors have had the same performance level, the probability to obtain such a result by chance (90% success vs 50%, respectively) was estimated to 2.7% by a two-tails bootstrap with 10^8^ iterations. Even though only a limited number of test cases was addressed in this article, this probability is low enough to clearly state that the ANOLEA/SSE performs better than ProsaII/SSE.

### Illustrative example

**Figure 8:**
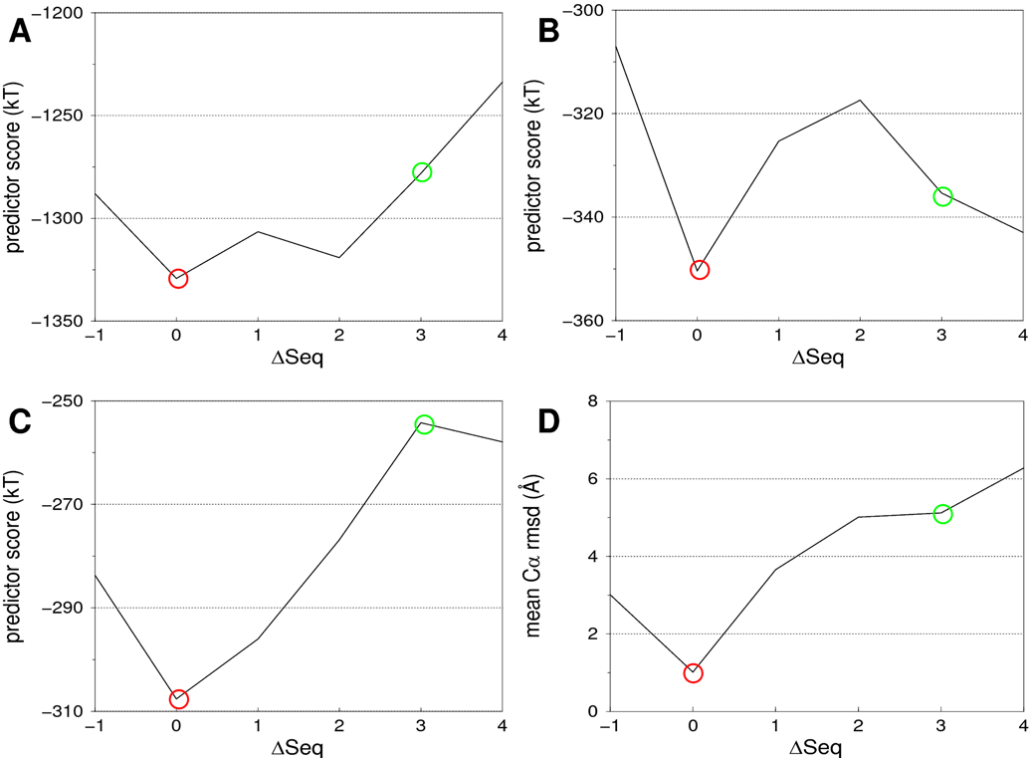
Impact of the distance between the evaluated alignment and the reference alignment (∶seq, X axis) on the three ANOLEA-based predictors (Y axis), for T0178 helix 7 case. The most favorable predictor score is indicated by a red circle and the structural alignment by a green circle. A, Predictor *ANOLEA/All*. B, Predictor *ANOLEA/ROI Local*. C, Predictor *ANOLEA/SSE Local*. Δ, Μεαν Χα RMSD in the region of interest of the models to crystal structure for each alignment.

The T0178 case from the CASP5 experiment was reported to be very difficult [35]. The sequence identity between the helix 7 of the template and the corresponding helix of the target is only 6%, and the global sequence identity is only 27%. A comparison between the reference alignment and the initial sequence alignment showed that the helix 7 of the latter was shifted by three residues toward the C-terminus (Fig. 3). For all alignments evaluated, the score assigned by the three ANOLEA-based predictors and the mean Cα RMSD between the models and the crystal structure are plotted in Figure 8. The structural alignment is identified correctly using the *ANOLEA/All*, the *ANOLEA/ROI* or the *ANOLEA/SSE* predictors (Fig. 8A, 8B and 8C, respectively). The two most favorable alignments according to the latter, corresponding to Δseq = 0 and Δseq = 1, are even separated enough to unambiguously point out the structural alignment, as reflected by a statistically significant p-value of 2.0⋅10^−2^.

### Impact of Δseq on final model quality

As expected, the closer to the structural alignment, the lower the RMSD to the X-ray structure of the resulting model. Figure 8D shows that the Cα RMSD in the ROI between the model and the crystal structure is about 1 Å when using the alignment identified by the *ANOLEA/SSE* predictor. This value rises quickly to 6 Å for incorrect alignments. Similar results are obtained for the other test cases (data not shown) .

## Discussion

The method presented in this paper makes use of structural information to refine misaligned regions between a sequence to model (the target) and its template. An exhaustive list of alternative ungapped alignments is generated, and their likelihood is evaluated in the structure space, using the following steps: 1) an ensemble of 100 models are generated from each alternative alignment using the MODELLER program, 2) each model is energy minimized with the CHARMM program, 3) the model quality is evaluated by predictor scores obtained using different pair wise energy functions (CHARMM, ProsaII and ANOLEA) computed on various residue selections around the misaligned region (*ALL, ROI, SSE*), 4) the alignments are ranked by statistically comparing the predictor score distributions of the corresponding models.

One important aspect that needed to be worked out for this approach to be successful is a careful accounting of the structural variability inherent to the ensemble of 100 models generated by satisfaction of spatial restraints. As illustrated in Figure 7, the distributions of the predictor values largely overlap, emphasizing the need to compare entire distributions rather than single values. In order to reach statistical significance while keeping the number of model low for CPU limitations, several aspects were considered; first, an energy minimization was added to refine models created by MODELLER. The standard deviation of the predictor value distribution was shown to be reduced in minimized models, as illustrated in Figure 5. This effect was more pronounced for ANOLEA and CHARMM based predictors which are more sensitive to small coordinate changes than ProsaII based predictors, data not shown. Second, the component of the noise resulting from distant parts of the structure was reduced by considering only the residues of the ROI and its neighbors, as illustrated by the improvements of correct predictions and their confidence when considering the *All* and the *ROI* subset of residues, see Table 2 and 3. Reducing further the variability by removing the contributions of the loops adjacent to the SSE led to a significant confidence improvement between the *ANOLEA/ROI* and the *ANOLEA/SSE* predictors, see Table 3.

The results of Table 3 alternatively show that MFP based predictors have a much higher success rate than semi-empirical force fields, and that taking into account the solvation free energy does not improve the reliability of the later. The performance of the predictors based on ANOLEA and ProsaII is similar for the *All* and *ROI* subset of residues, but when considering the *SSE* subset of residue, ANOLEA clearly outperforms ProsaII (90% success versus 50%). The good performance obtained with of the *SSE* subset of residue comes from its ability to limit the impact of the high variability inherent to homology modeling techniques.

These data suggest that the conformation of a SSE in its local protein environment does correspond to a local minimum of its free energy. Although this result is somewhat intuitive, it is not an universal property; the conformation of particular regions of a protein might be constrained by the rest of the fold so that the minimum free energy conformation of the total protein results in SSE conformations that are driven away from their local minima. Experimental evidences also support this idea; pieces of a cleaved protein can recombine via non covalent interactions to form a structure with properties very similar to the native ones, while the separated fragments alone are devoid of any structure and function [36], [37]. In the case of the bovine RNAse S [36], which shares a common fold with the EDN test case that was optimized, the fragment is a stretch of 20 residues at the N-terminal part of the protein containing an 8 residues long helix flanked by two loops. This helix of the RNAse S fragment corresponds to the helix 1 of the EDN test case. Additional experiments on RNAse S have also shown that even after the deletion of 5 loop residues in the C-terminal part of the fragment, the complex still conserves its function and stability [38]. The same observation was reported for the staphylococcal nuclease [37], where the protein is cleaved in a first 43 residues fragment containing 4 strands and a second 100 residues fragment (with 4 strands and 3 helices). As stated by Anfinsen et al. [39], “the cleavages and deletions do not destroy the geometric “sense” of the chain”. These observations suggest that the interaction between secondary structure elements and their local environment is determinant for the stability of a protein. In our approach, the success of the *SSE* predictor compared to *All* and *ROI* predictors comes as an illustration of this general principle.

### Conclusions

The alignment between a target and its template is a current bottleneck in homology modeling approaches, and methodological improvements are needed to overcome this limitation, especially when sequence identity is low. Although energy-based methods are widely used to tackle this problem, they are currently limited by their accuracy. This study shows that a small number of high-quality, all atom, and minimized models are sufficient to reliably evaluate a single alignment when using a sensitive and accurate scoring function. The reliability of the prediction is greatly enhanced by considering only the SSE to optimize and its interaction with residues of adjacent SSEs only, neglecting the loops, whose large structural variability adds noise and impairs the prediction based on the total energy

The method proposed in this article is able to discriminate the structural alignment from several alternatives. Its success mainly depends on the template quality in the ROI and its surrounding. If the orientation, length and environment of misaligned secondary structure are similar between the target and the template, the approach has shown to be very efficient. The proposed sampling method explores the free energy landscape of a SSE with the assumption that no gaps are present. A full alignment optimization combining an enhanced variant of the *ANOLEA/SSE* predictor (for scoring) and a genetic algorithm (for sampling) is currently under investigation.
